# Unique temperature change patterns in calves eyes and muzzles: a non-invasive approach using infrared thermography and object detection

**DOI:** 10.3389/fvets.2025.1548906

**Published:** 2025-03-10

**Authors:** Sueun Kim, Norio Yamagishi, Shingo Ishikawa, Shinobu Tsuchiaka

**Affiliations:** Laboratory of Large Animal Clinical Medicine, Graduate School of Veterinary Sciences, Osaka Metropolitan University, Osaka, Japan

**Keywords:** infrared thermography, non-invasive temperature measurement, AI object detection, temperature change patterns, cattle welfare monitoring

## Abstract

This study investigates the potential of non-invasive, continuous temperature measurement techniques for assessing cattle welfare. We employed advanced object detection algorithms and infrared thermography to accurately extract and continuously measure temperatures of the eyes and muzzles of 11 calves over several months (total, 33 samples). A mobile thermal imaging camera was paired with the Mask R-CNN algorithm (object detection) trained on annotated datasets to detect eye and muzzle regions accurately. Temperature data were processed by outlier rejection, standardization, and low-pass filtering to derive temperature change patterns. Cosine similarity metrics and permutation tests were employed to evaluate the uniqueness of these patterns among the individuals. The average cosine similarity between eye and muzzle temperature changes in the same individual across 33 samples was 0.72, with permutation tests yielding *p*-values <0.01 for most samples, indicating pattern uniqueness. This study highlights the potential of high-frequency, non-invasive temperature measurements for detecting subtle physiological changes in animals without causing distress.

## 1 Introduction

Temperature is measured routinely in clinical settings and is a crucial diagnostic tool for assessing an animal’s health status and detecting potential illnesses or physiological changes. The most common and traditional method is rectal temperature measurement. However, sometimes this method can be stressful, which has prompted investigations into alternative, non-invasive techniques. Recent studies have explored the correlation between eye temperature, measured using infrared cameras, and rectal temperature in various species, including cats (1), sheep (2), and cattle (3, 4). These studies have demonstrated medium to strong correlations, suggesting that eye or muzzle temperature measurements could serve as viable substitutes for core body temperature measurements. Despite the advancements in measurement techniques, the current infrared camera-based temperature measurement techniques have limitations. The region of interest (ROI) in the images is typically defined using rectangular or circular shapes (1–3, 5, 6), which may not allow for precise extraction of the desired area. Furthermore, manual temperature measurements result in discontinuous data collection. Indeed, previous studies have consistently employed intermittent measurement protocols, with temporal intervals between data points (2, 4, 7, 8).

Recent advancements in artificial intelligence have led to its widespread application in scientific fields. A recent study combining object detection with infrared camera technology for respiratory pattern analysis (9) demonstrated the following two key advantages: (1) accurate ROI identification and (2) continuous temperature measurements at a rate of 8.7 frames/s. Regarding accurate ROI identification, this will help overcome the limitation of infrared camera measurements mentioned above. Moreover, continuous temperature measurement allows for consistent tracking of eye and muzzle temperatures, potentially opening up new research possibilities. At this point, establishing the reliability and validity of continuous measurement methods is crucial. Therefore, this study aims to substantiate these claims through two distinct processes. First, temperatures were accurately and continuously measured from different areas (eyes and muzzle) in cattle to determine if similar patterns of temperature change were observed in the same individual in continuous cases. Second, temperature change patterns from randomized patterns were compared to identify whether the characteristics of short-term, continuously measured temperature change patterns are unique.

## 2 Materials and methods

### 2.1 Animals and devices

For this study, data from 11 calves (aged 12–14 weeks) were collected and analyzed at the Kobe University farm (Food Resources Education and Research Center, Graduate School of Agricultural Science Kobe University). Each calf underwent a series of thermal imaging sessions at four-week intervals, including three separate imaging events, from February to September (ambient temperature range: 9°C–34.1°C). This systematic approach yielded a comprehensive dataset comprising 33 distinct imaging samples. Imaging was conducted between 11:00 and 13:00 during the post-feeding rumination period. To minimize disturbance, the researchers approached the calves slowly and conducted imaging from a distance of 1 m. Data collection included both thermal imaging using an infrared camera and conventional video recording (1–2 min) using a standard camera attached to an infrared camera device. The calves were housed at the Kobe University farm in a semi-open facility, characterized by one side being completely open to the external environment.

We used a mobile thermal imaging camera (FLIR One Pro for iOS [accuracy: ±3.0°C, sensitivity: 0.07°C, field of view: 55° × 43°, resolution: 80 × 60, temperature range: −20°C–400°C], FLIR Systems Inc., Santa Barbara, CA, USA).^1^ The emissivity was set to 0.95 (the correct emissivity for animal tissue is 0.98; however, it was set to 0.95 due to the limitation of the device’s emissivity setting).

This study was approved by the Institutional Animal Care and Use Committee of the Osaka Metropolitan University (approval number: 24–024).

### 2.2 Image extraction and temperature derivation

Image extraction and temperature derivation were performed according to the protocols established in previous research (9).

#### 2.2.1 Algorithm training for image extraction

Algorithm training for image extraction denotes a series of methodological steps (annotation, training, and inference) designed to enable the identification and isolation of specific features (namely, eyes and muzzle) in novel red, green, and blue (RGB) images.

Annotation: the process of creating a dataset (annotated images) for training. Annotated images are those that have labels added to highlight specific features of interest. These annotations may include bounding boxes, lines, arrows, or text indicate and describe specific regions within the image. In this study, we used lines to mark the relevant features (eyes or muzzles). We annotated eye and muzzle regions in 1000 images (700 for training and 300 for validation) using the VGG Image Annotator (VIA) (10). With the annotated images as dataset, the training models can later perform tasks like object detection, segmentation, and classification.

Training: it involves algorithms to locate objects in images through annotated datasets and iterative learning. As for training algorithm, we used the Mask R-CNN architecture (11) in conjunction with transfer learning, utilizing a pre-trained model for detection training. Transfer learning enhances learning outcomes by leveraging knowledge acquired from a previously mastered, related task. This approach is particularly beneficial when the available dataset is insufficient for comprehensive training, allowing the use of a model trained on a similar, existing dataset. Our dataset, comprising 1,000 images, was deemed inadequate for full-scale training. Consequently, we used the COCO (Common Objects in Context) dataset (12), which is a large-scale object detection dataset developed by Microsoft. The COCO dataset, which includes various animal categories, shared similarities with our target domain. We leveraged a pre-trained backbone weight based on the COCO dataset to expedite the training process. For the backbone architecture, we implemented ResNet 101 (13). By combining ResNet 101 with the COCO dataset, we fine-tuned a new classifier specifically for this eye and muzzle detection task. This process involved adjusting the model’s weights.

Inference: the process where a trained model localizes objects in new images with weights. Weights in neural networks are numerical values that determine how strongly different features influence the model’s decisions. During training, these weights are iteratively updated to improve the model’s ability to recognize specific patterns, in this case, eyes and muzzles. Using these optimized weights, the model can then effectively locate regions of interest in new, unseen images.

#### 2.2.2 Temperature change pattern derivation

To determine temperature variation patterns, we obtained (1) temperature sequence data from the infrared camera and (2) conventional video images from the standard camera for 33 samples. The conventional video images were processed using the previously obtained weights to extract eye and muzzle regions. The identified eye and muzzle locations were then used to extract the corresponding temperature data from the temperature sequence data. Finally, mean temperatures were calculated to determine eye and muzzle temperatures. This process was repeated for all frames to derive time-dependent temperature changes in the eyes and muzzles.

To ensure the reliability and validity of our temperature variation analysis, we developed a comprehensive data processing protocol. It involved a multi-step approach to ensure data integrity and comparability. Initially, we implemented an outlier rejection protocol utilizing Tukey’s hinge method (*g* = 1.5) (14), which is a widely accepted technique in statistical analysis. This method defines an acceptable range based on the following formula:


$$\left[Q_{1}-g\left(Q_{3}-Q_{1}\right),Q_{3}+g\left(Q_{3}-Q_{1}\right)\right]$$

where *Q*_1_ and *Q*_3_ represent the first and third quartiles of temperature distribution, respectively, and *g* denotes Tukey’s constant. Subsequently, to address the variability in absolute temperature measurements stemming from inter-individual differences and environmental factors, we applied a standardization procedure to the data. This step facilitates more meaningful comparisons across subjects and conditions. Finally, the graph incorporates various sources of noise, including camera frame artefacts, external environmental factors such as wind, and physiological interferences stemming from cardiac and respiratory activities. To mitigate these diverse noise elements, a low-pass filtering technique was employed, with a cut-off frequency set at 0.08 Hz. This methodological sequence ensures robust data preprocessing, accounting for outliers, individual variability, and measurement-induced fluctuations, thereby providing a solid foundation for subsequent analysis of temperature change patterns.

### 2.3 Statistical analysis

#### 2.3.1 Cosine similarity

To analyze and compare the temperature change patterns of two distinct graphs, we employed cosine similarity as our primary metric. This approach allows the comparison of graph structures. The temperature change patterns of the eye and muzzle, extracted from the same individual, were represented as vectors. Subsequently, the cosine similarity between these two vectors was calculated using the following formula:


$$\mathrm{cosine}\;\mathrm{similarity}=\frac{V1\cdot V2}{∥V1∥∥V2∥}$$

The resulting cosine similarity value, ranging from −1 to 1, was interpreted as follows: values closer to 1 indicate high similarity in variability patterns, values closer to 0 suggest orthogonality or dissimilarity, and negative values, if present, indicate inverse relationships.

#### 2.3.2 Permutation test

To assess the statistical significance of the observed similarities, we conducted a permutation test (15) with 10,000 iterations. This procedure included the following steps: (1) calculation of the observed cosine similarity between the two graphs (the temperature change patterns of the eye and muzzle, extracted from the same individual), (2) random permutation of the elements of one of the vectors (the temperature change patterns of the muzzle) multiple times (10,000 iterations), (3) calculation of cosine similarity with the unpermuted vector (the temperature change patterns of the eye) for each permutation. The number of permuted similarities ≥ the observed similarity was recorded. This value was then divided by the total number of permutations (10,000) to derive the *p*-value. This analysis provided a *p*-value to quantify the likelihood of observing such similarity by chance. All computations and analyses were performed using Python 3.8 with the NumPy and SciPy libraries. The visualizations were generated using Matplotlib.

## 3 Results

**Figure 1:**
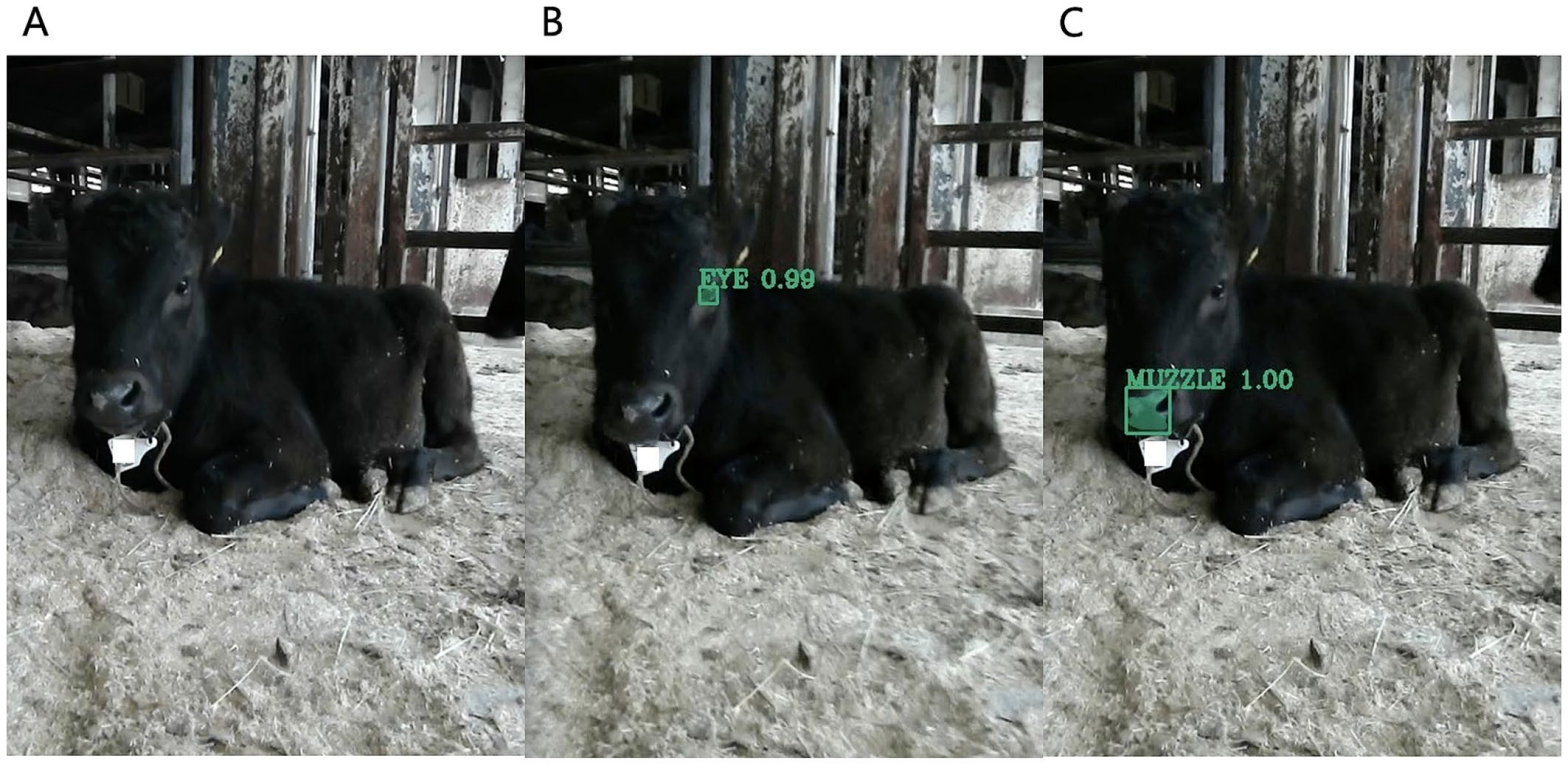
Results of using object detection to identify calves eye and muzzle. **(A)** Image of a calf captured using a standard camera. **(B)** Image of the eye region extracted by object detection. **(C)** Image of the muzzle region. The green-bound boxes represent object detection, while the green hatched areas denote pixel-wise object detection, also known as segmentation.

By training utilizing annotated images and the Mask R-CNN algorithm, we obtained weights that enabled the detection of eyes and muzzles in the images captured for this study (Figure 1).

**Figure 2:**
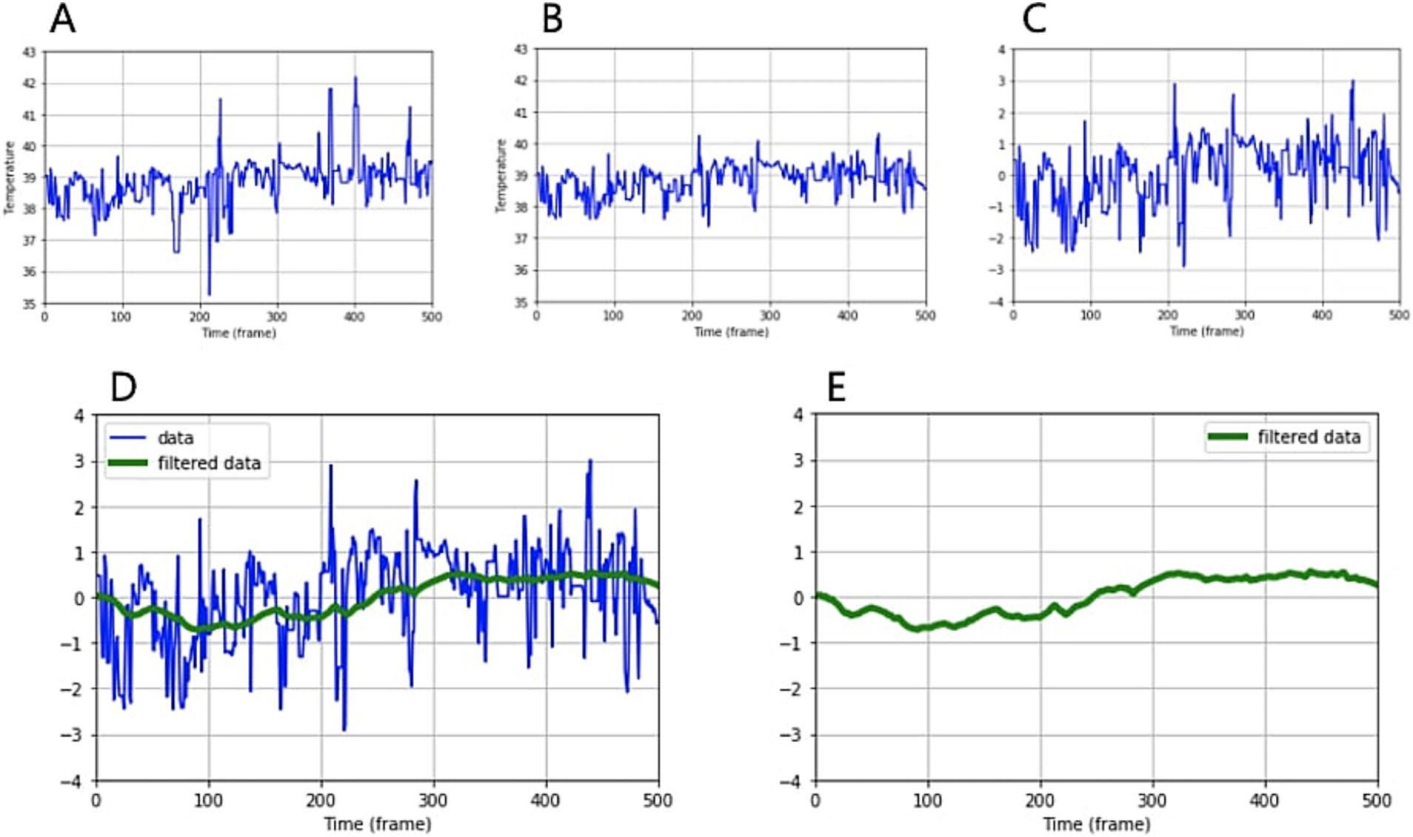
Analyzing and processing temperature data over time. **(A)** Temperature graph over time obtained by combining infrared temperature data and object extraction **(B)** after outlier rejection, **(C)** after standardization, **(D)** after low-pass filtering. The green line represents the filtered graph. **(E)** A pattern of temperature change over time.

Subsequently, by combining temperature data from infrared cameras with the location data of eyes and muzzles extracted from standard images, we were able to derive temperature changes over time. After applying outlier rejection, standardization, and low-pass filtering, we obtained temperature change patterns for the eyes and muzzles (Figure 2).

**Figure 3:**
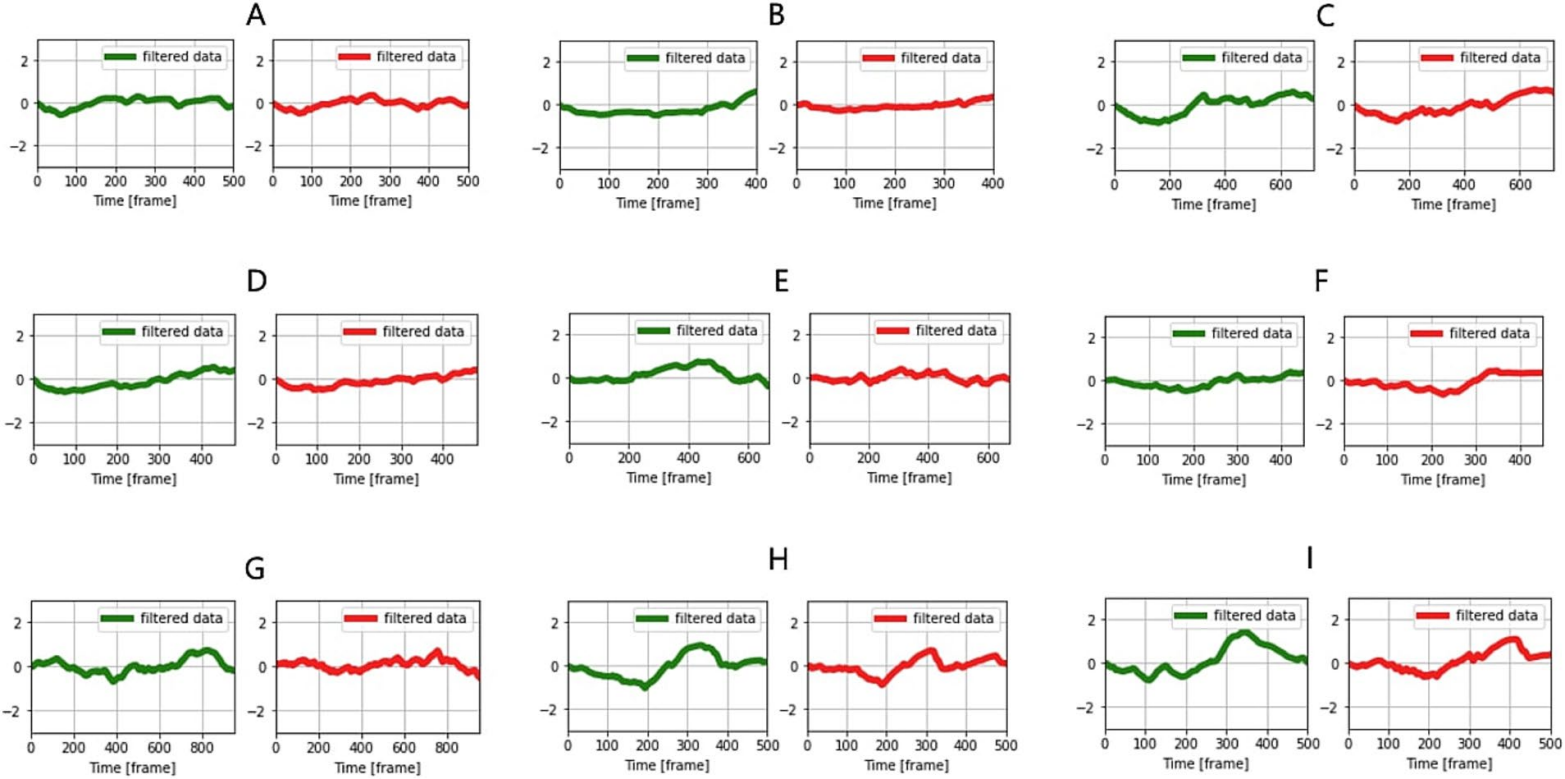
Comparison of temperature change patterns in the eye and muzzle in the same individual **(A–I)**. Green indicates eye and red, muzzles.

The average cosine similarity obtained by comparing the similarity of eye and muzzle temperature changes in the same individual across 33 samples (Figure 3) was 0.72 (Table 1).

**Table 1:** Cosine similarity among samples.

Cosine similarity	Number of samples	*p*
0.5–1	26	*p* < 0.01
0.3–0.5	4	*p* < 0.01
<0.3	3	*p* > 0.5

A permutation test, which involved randomizing one sample (muzzle temperature change) in the same individual and comparing its similarity with non-randomized samples (eye temperature change), yielded a *p*-value <0.01 (29 of 33 samples). This was interpreted to mean that randomized graphs could not exhibit high cosine similarity, and the original graph patterns possessed uniqueness. However, 4 of 33 samples showed cosine similarities close to 0 and *p*-values >0.5 in the permutation test. For these 4 samples, difficulties in detecting eyes in the captured images were observed, resulting in the complete failure to detect the eyes or erroneous identification of other parts (skin or body parts). Additionally, 3 samples failed to exceed a cosine similarity of 0.5. In these cases, the calves’ heads were turned sideways, and the muzzle portions in the images were small with prominently captured nostrils, suggesting that the muzzle temperatures were relatively strongly influenced by respiration.

## 4 Discussion

In this study, we employed object detection, an AI technology, to enhance the accuracy of ROI detection. We also utilized temperature data from all frames of the infrared camera to obtain high-frequency temperature change variability. The reliability of this method was verified by comparing the variability between two areas (eye and muzzle). This results confirmed that short-term temperature changes (1–2 min) exhibit unique characteristics. Furthermore, distinctive temperature variations were identified by measuring eye and muzzle temperatures. However, muzzle measurements might have been influenced by the sideways movement of the calf’s head, resulting in a smaller muzzle area in the image, or strong respiration. Notably, this study was conducted in a field setting rather than a controlled environment, thus validating its potential for practical application in real-world scenarios.

### 4.1 Utilization of temperature measurements

Research utilizing Infrared Thermography (IRT) to detect physiological changes in animals is increasingly supported by studies across diverse species and contexts. For instance, IRT has been validated as a tool to identify heat stress in farm animals by detecting vasodilation-mediated temperature changes in thermal windows like the ocular region and muzzle (16). Additionally, IRT has been integrated with heart rate variability (HRV) metrics to comprehensively assess autonomic nervous system (ANS) activity during stress responses (17). In clinical applications, IRT’s non-invasive nature has proven valuable for monitoring ischemic events and tracking inflammatory responses during wound healing (18). Recent advances also highlight its utility in studying temporal body temperature dynamics. For example, infrared technology has been used to quantify circadian rhythmicity (24-h cycles) (19) and short-term fluctuations (~10-min intervals) in core body temperature (20). Notably, avian studies demonstrate rapid stress-induced thermal responses, such as transient eye temperature drops followed by rebounds within 30 s, underscoring the need for sub-second measurement resolution to capture acute physiological shifts (21, 22). Accordingly, in this study, we aimed to measure temperature at a frequency of less than 1 s using temperature data from all consecutive frames of the infrared camera. We were able to obtain reliability through measurement and comparison at two locations: the eyes and muzzle. The implementing high-frequency temperature measurements at intervals of ≤1 s, as employed in this study, represents a significant methodological advancement in animal welfare monitoring. This approach is comparable to the difference between simple heart rate measurement and continuous heart rate monitoring that enables HRV analysis, revealing information that would otherwise be undetectable. Continuous data collection allows for various mathematical calculations, and consequently, its improved sensitivity might detect subtle temperature fluctuations, potentially revealing minor stressors or physiological changes imperceptible with less frequent sampling. In fact, a human study (23) has successfully detected stress through continuous measurement of nasal tip temperature and various mathematical analyses of this data, such as temperature difference between from the start and the end, slope of thermal variable signal, and standard deviation of successive differences of thermal variable signal. The current study has verified the validity and reliability of temperature changes in calves’ eyes and muzzles, which we believe will serve as a foundation for diverse analyses in future research.

### 4.2 Object detection-based measurements

Recent advancements in AI have led to its integration with existing technologies, rather than relying solely on AI, enabling us to overcome the limitations of traditional devices. A prime example of this synergy is the integration of AI with infrared cameras in various studies. For instance, in bovine research, AI has been successfully integrated with infrared camera imagery to detect digital dermatitis (24), mastitis (25), respiratory patterns (26), and body temperature (27). These applications demonstrate how AI can augment the analytical power of thermal imaging. While the aforementioned integrations typically use AI to identify specific areas of interest within infrared images, our study takes a different approach. We aim to address the inherent limitations of infrared imaging by using object detection techniques on RGB images. This novel approach allows us to overcome some of the constraints associated with thermal imaging alone. To elaborate further on our methodology, traditional infrared camera-based methods of measuring eye or muzzle temperatures were limited by rectangular or circular ROI settings (5) and manual configuration requirements. These limitations often resulted in the inclusion of surrounding skin areas within the ROI, potentially leading to inaccurate temperature readings due to temperature differentials between the eye and surrounding skin. Previous studies (3) showing only medium correlations between rectal and muzzle temperatures may be attributed to imprecise muzzle area extraction. This study, however, employed object detection techniques, specifically utilizing the segmentation capabilities of Mask R-CNN. This approach enabled precise pixel-level detection of eye and muzzle regions, consequently enhancing the accuracy of temperature calculations, which in turn provided a more robust foundation for analyzing thermal variation patterns in cattle.

Despite these advancements, a significant challenge in our study’s AI-based detection of bovine eyes and muzzles lies in distinguishing the eyes within the cranial region. This difficulty arises from the chromatic similarity between the eye and surrounding fur. However, the detection process employed in this study proved successful in a majority of the cases. Despite this success, four samples yielded near-zero similarity scores, likely due to detection failures, which can be attributed to external factors, such as backlighting and overcast weather conditions. It is noteworthy that under such circumstances, even human visual discrimination of the eye region would be considerably challenging.

### 4.3 Field measurements

There are numerous constraints related to infrared camera temperature measurements in field settings. External environmental factors can influence measured temperatures, with cold or windy conditions often resulting in lower temperature readings (28, 29). In this study, measurements were taken across various seasons (February to September, ambient temperature range: 9°C–34.1°C), revealing a wide range of temperatures. Additionally, it is known that infrared camera temperature readings can vary due to individual factors such as skin thickness and hair density (30), differences in infrared emissivity based on color variations (31), and breed-specific characteristics (32). Indeed, in this study, we were able to observe temperature differences between individuals. The continuous temperature measurements and the resulting temperature variability analysis in this study are significant in their potential for standardization, which could help overcome the aforementioned limitations. Standardization allows us to focus on relative changes over time rather than absolute values. This approach is expected to minimize the differences in absolute temperatures caused by external environmental factors and individual variations among animals, thereby allowing for a clearer focus on physiological changes of interest. This approach adds significant value to the research, enabling more reliable analyses of temperature variability in animals, despite the inherent challenges of field-based infrared thermography.

## 5 Conclusion

This study demonstrates the efficacy of combining artificial intelligence-based object detection with infrared camera technology for continuous, non-invasive temperature measurement in cattle. The research yielded several significant findings. First, short-term temperature changes (1–2 min) in cattle exhibit unique characteristics, as evidenced by the high average cosine similarity (0.72) between eye and muzzle temperature patterns in the same individual. Second, the permutation test results (*p* < 0.01) confirm that these temperature change patterns possess distinctive features that cannot be replicated by random fluctuations. Finally, this study’s field setting validates its potential for practical application in real-world scenarios. The methodology developed here offers a promising approach for enhancing animal welfare monitoring in field conditions, potentially enabling early detection of stress or health issues in livestock.

## Data Availability

The raw data supporting the conclusions of this article will be made available by the authors, without undue reservation.

## Footnotes

^1^ www.flir.com
